# Quality of life and neurological symptoms in patients with neurofibromatosis type 2: a national database study in Japan

**DOI:** 10.1265/ehpm.24-00158

**Published:** 2024-08-28

**Authors:** Takashi Yamauchi, Machi Suka

**Affiliations:** Department of Public Health and Environmental Medicine, The Jikei University School of Medicine, 3-25-8 Nishi-shimbashi, Minato-ku, Tokyo 105-8461, Japan

**Keywords:** Rare disease, Neurofibromatosis type 2, Quality of life, Medical certificate, National database

## Abstract

**Background:**

This study examined the association between neurological symptoms and quality of life (QoL) in patients with neurofibromatosis type 2 (NF2) using a national database of all patients who newly claimed for medical expense subsidies in Japan from 2015 to 2019.

**Methods:**

The Japanese Ministry of Health, Labour and Welfare provided access to the “National Database of Designated Intractable Diseases of Japan” containing the “Medical Certificates of Designated Intractable Diseases” of all patients with NF2. The database included information on five items of QoL: “mobility,” “self-care,” “usual activities,” “pain/discomfort,” and “anxiety/depression.” To examine the association between the presence/absence of neurological symptoms and QoL, multivariable logistic regression analyses were performed, adjusted for potential confounders.

**Results:**

Data from 187 patients (97 females and 90 males; mean (standard deviation) age, 43.1 (17.9) years) were analyzed. Overall, 31% to 55% of patients were recorded as having moderate/severe impairment of QoL. Spinal dysfunction was significantly associated with deterioration of all components of QoL, whereas speech dysfunction and hemiparesis were specifically associated with physical health-related components of QoL. Spinal dysfunction, facial nerve palsy, and age 25–64 years were significantly associated with “anxiety/depression.”

**Conclusions:**

In the present epidemiological study using a national database of NF2 in Japan, spinal dysfunction was significantly associated with deterioration of all components of QoL, including subjective and mental health-related components of QoL, whereas speech dysfunction and hemiparesis were specifically associated with physical health-related components of QoL.

**Supplementary information:**

The online version contains supplementary material available at https://doi.org/10.1265/ehpm.24-00158.

## Graphical Abstract

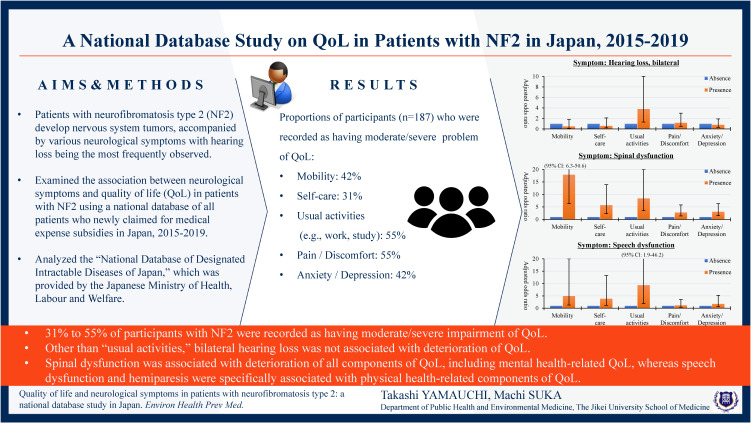

## Background

Neurofibromatosis type 2 (NF2) is a rare autosomal-dominant neurocutaneous disease resulting from mutations in the NF2 tumor suppressor gene. An estimated birth incidence of NF2 is approximately one in 25,000 [1, 2]. Patients with NF2 develop nervous system tumors, including schwannomas, meningiomas, and neurofibromas [1, 2], accompanied by various neurological symptoms with hearing loss being the most frequently observed [3]. There is currently no established treatment for NF2, and thus enhancing/maintaining quality of life (QoL) is an important issue for patients with NF2.

Previous systematic reviews on the QoL of patients with rare genetic skin diseases, including NF2, have highlighted the impact of these diseases on general QoL and physical/psychological functioning [4, 5]. However, studies included in those reviews had small sample sizes [4], especially those pertaining to NF2 (e.g., n = 62) [6], and were conducted only in North American or European countries [5]. To our knowledge, no study has investigated the state of QoL among patients with NF2 in Asian countries including Japan.

Japan has implemented comprehensive measures against intractable rare diseases [7]. In January 2015, the Intractable Rare Disease Act (IRDA) was enacted to promote financial support for patients suffering from rare diseases in Japan. The diagnostic criteria and clinical stage classification for each rare disease have been established to standardize the criteria for medical expense subsidies [8]. To receive medical expense subsidies, patients with rare diseases, including NF2, must submit a claim to the prefectural government, along with a “Medical Certificate of Designated Intractable Diseases” filled out by a physician. The Japanese Ministry of Health, Labour and Welfare (MHLW) has created a database of these medical certificates. It has been reported that approximately 800 patients have claimed medical expense subsidies for NF2 at least once between 2009 and 2013 [9].

Using this database, some epidemiological studies have examined the social independence [10, 11] and characteristics of progressive disabilities [12] in patients with NF2 in Japan. However, those studies did not assess the levels of QoL in patients with NF2 because five assessment items for QoL were added to the medical certificate after the 2015 enactment of the IRDA.

Since 2015, the MHLW has established a system in which the new nationwide database (“National Database of Designated Intractable Diseases of Japan”) of patients with rare diseases could be provided to researchers for research purposes following approval of study protocols [13]. Using this database, the present epidemiological study aimed to examine the association between the presence/absence of neurological symptoms of NF2 and QoL in patients with NF2. A better understanding of QoL among patients with NF2 based on a national database may contribute to the promotion of physical/psychological well-being, such as improvement of daily living, social participation/involvement, and coexistence with the disease, in this patient population.

## Methods

### Data source

The MHLW approved the study protocol and provided the database that included information about all patients who newly claimed for medical expense subsidies for NF2 from 2015 to 2019. The MHLW removed all personally identifiable information from the database.

In Japan, the diagnosis of NF2 is made by physicians according to the criteria set forth by the National Institutes of Health [14]. Patients with at least one of the following neurological symptoms of NF2 are eligible for medical expense subsidies: hearing loss, facial nerve palsy, cerebellar dysfunction, decreased facial sensation, speech dysfunction, double vision, blindness, hemiparesis, seizures, and spinal dysfunction. Following a previous study [11], dysphagia or dysarthria and aphasia were included in “speech dysfunction.” The clinical stage and severity of neurologic disability of NF2 were assessed for each patient using a 25-point scoring system [12, 15].

**Fig. 1:**
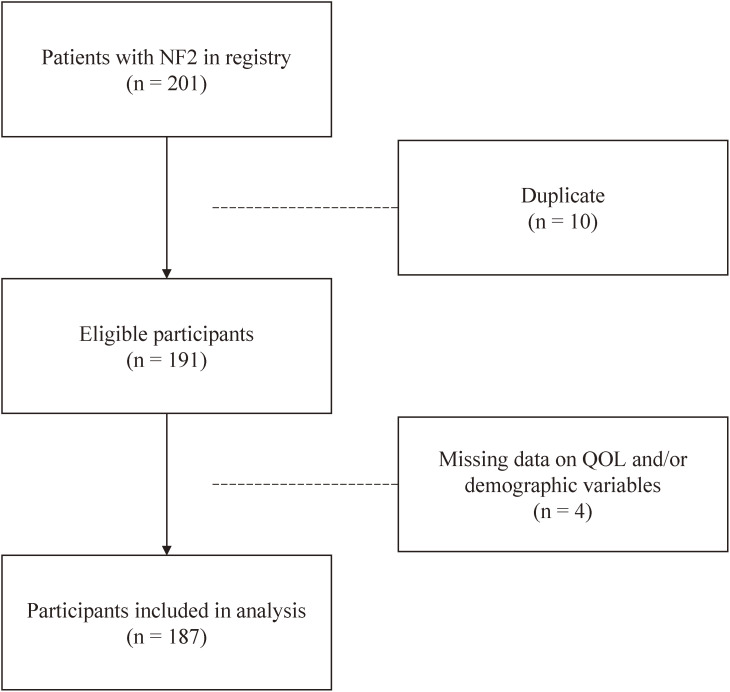
Flowchart of study participant selection

During 2015–2019, the database included data on 201 patients with NF2 who newly submitted claims to receive medical expense subsidies (Fig. 1). Eligibility criteria were: (1) new registrants during 2015–2019 and (2) no duplicate data.

The study protocol was approved by the institutional review board of the Jikei University School of Medicine (No. 33-045(10655)). Results of the present study were obtained from analyses conducted specifically for research purposes using the National Database of Designated Intractable Diseases of Japan and thus do not correspond to the relevant statistics publicized by the MHLW.

### Variables

The database included the following information: (1) year at registration, (2) presence/absence of neurological symptoms of NF2, (3) clinical stage of NF2, (4) family history of NF2, (5) QoL, and (6) demographic variables (i.e., sex and age).

QoL was assessed using five items – “mobility,” “self-care,” “usual activities,” “pain/discomfort,” and “anxiety/depression” – which were adopted by the MHLW (Table 1). Regarding the five components of QoL, the MHLW requests that these items be used separately (i.e., not combined into a single indicator) when assessing QoL. Accordingly, each of the five QoL components was examined separately in statistical analyses.

**Table 1:** Quality of life among participants (n = 187)

	**n**	**(%)**
Mobility		
M0: I have no problem walking about	108	(58)
M1: I have some problems walking about	69	(37)
M2: I am confined to bed	10	(5)
Self-care		
S0: I have no problem with self-care	129	(69)
S1: I have some problems washing or dressing myself	46	(25)
S2: I am unable to wash or dress myself	12	(6)
Usual activities (e.g., work, study, housework, family or leisure activities)		
U0: I have no problem performing my usual activities	83	(44)
U1: I have some problems performing my usual activities	94	(50)
U2: I am unable to perform my usual activities	10	(5)
Pain / Discomfort		
PD0: I have no pain or discomfort	84	(45)
PD1: I have moderate pain or discomfort	88	(47)
PD2: I have extreme pain or discomfort	15	(8)
Anxiety / Depression		
AD0: I am not anxious or depressed	109	(58)
AD1: I am moderately anxious or depressed	62	(33)
AD2: I am extremely anxious or depressed	16	(9)

### Statistical analysis

First, cross-tabulation between the presence/absence of neurological symptoms and clinical stage of NF2 and QoL was performed. Following a request from the MHLW, we removed data from Tables and Appendices if the number of patients corresponding to the data was less than 10.

To examine the association between the presence/absence of neurological symptoms and clinical stage of NF2 and each QoL component adjusted for potential confounders (i.e., age and sex), multivariate logistic regression analyses were performed with “no problem” for each QoL component (i.e., M0, S0, U0, PD0, and AD0 in Table 1) as the reference category of the dependent variable. The proportional odds assumption for QoL was not met, and the number of participants included in each category of QoL components was relatively small. Thus, we did not perform ordinal or multinomial logistic regression analyses, and combined categories other than “no problem” for each QoL component. Adjusted odds ratios (ORs) and 95% confidence intervals (CIs) were calculated.

P < 0.05 was considered statistically significant. All analyses were performed using SPSS version 25 (IBM, Chicago, IL, USA).

## Results

Of the 201 newly registered patients during the 5-year study period, 10 were ineligible for inclusion due to duplicate data, and 4 were excluded from analyses due to missing data. The final study population consisted of 187 participants (97 females and 90 males; mean (SD) age at registration, 43.1 (17.9) years) (Fig. 1).

Table 2 summarizes participant characteristics. The number of participants presenting with each neurological symptom varied widely by symptom; 28% and 27% had unilateral and bilateral hearing loss, respectively, and 41% had spinal dysfunction. Nearly 47% of participants were classified as Stage 4.

**Table 2:** Participant characteristics (n = 187)

	**n**	**(%)**
Year of claim for medical expense subsidies		
2015	24	(13)
2016	29	(16)
2017	49	(26)
2018	38	(20)
2019	47	(25)
Sex		
Male	90	(48)
Female	97	(52)
Age		
0–24 years	37	(20)
25–44 years	63	(34)
45–64 years	62	(33)
>64 years	25	(13)
Family history of NF2		
Present	45	(24)
Absent	102	(55)
Unknown	37	(20)
Presence of symptoms		
Hearing loss, unilateral	52	(28)
Hearing loss, bilateral	51	(27)
Facial nerve palsy	38	(20)
Cerebellar dysfunction	39	(21)
Decreased facial sensation	36	(19)
Speech dysfunction	38	(20)
Double vision	18	(10)
Blindness	11	(6)
Hemiparesis	18	(10)
Seizures	11	(6)
Spinal dysfunction	76	(41)
Clinical stage		
Stage 0	32	(17)
Stage 1	21	(11)
Stage 2	35	(19)
Stage 3	11	(6)
Stage 4	88	(47)

Proportions of participants who were recorded as having no problem for each QoL component (i.e., M0, S0, U0, PD0, and AD0 in Table 1) ranged from 44% (“usual activities”) to 69% (“self-care”). On the other hand, participants who were recorded as having severe impairment in any QoL component (i.e., M2, S2, U2, PD2, and AD2 in Table 1) accounted for less than 10%.

Table 3 summarizes the results of cross-tabulation between the presence/absence of neurological symptoms and each QoL component. The presence of neurological symptoms was generally associated with the deterioration of several QoL components. All patients with hemiparesis (n = 18) experienced moderate or severe impairment of “mobility” and “usual activities.”

**Table 3:** Cross-tabulation between the presence of neurological symptoms and quality of life (QoL) (n = 187)

**QoL component^a)^**	**Mobility**				**Self-care**				**Usual activities**				**Pain / ** **Discomfort**				**Anxiety / Depression**			
					
	**M0**		**M1/M2**		**S0**		**S1/S2**		**U0**		**U1/U2**		**PD0**		**PD1/PD2**		**AD0**		**AD1/AD2**	
									
	**n**	**(%)**	**n**	**(%)**	**n**	**(%)**	**n**	**(%)**	**n**	**(%)**	**n**	**(%)**	**n**	**(%)**	**n**	**(%)**	**n**	**(%)**	**n**	**(%)**
Presence of symptoms^b,c)^																				
Hearing loss, bilateral (n = 52)	32	(62)	20	(38)	37	(71)	15	(29)	25	(48)	27	(52)	28	(54)	24	(46)	29	(56)	23	(44)
Hearing loss, unilateral (n = 51)	26	(51)	25	(49)	29	(57)	22	(43)	12	(24)	39	(76)	17	(33)	34	(67)	27	(53)	24	(47)
Facial nerve palsy (n = 38)	15	(39)	23	(61)	19	(50)	19	(50)	12	(32)	26	(68)	16	(42)	22	(58)	15	(39)	23	(61)
Cerebellar dysfunction (n = 39)	—		—		15	(38)	24	(62)	—		—		14	(36)	25	(64)	16	(41)	23	(59)
Decreased facial sensation (n = 36)	12	(33)	24	(67)	14	(39)	22	(61)	—		—		—		—		15	(42)	21	(58)
Speech dysfunction (n = 38)	—		—		12	(32)	26	(68)	—		—		12	(32)	26	(68)	13	(34)	25	(66)
Double vision (n = 18)	—		—		—		—		—		—		—		—		—		—	
Blindness (n = 11)	—		—		—		—		—		—		—		—		—		—	
Hemiparesis (n = 18)	—		—		—		—		—		—		—		—		—		—	
Seizures (n = 11)	—		—		—		—		—		—		—		—		—		—	
Spinal dysfunction (n = 76)	21	(28)	55	(72)	35	(46)	41	(54)	14	(18)	62	(82)	20	(26)	56	(74)	32	(42)	44	(58)

a) Classification of each component

Mobility

M0: I have no problem walking about

M1: I have some problems walking about

M2: I am confined to bed

Self-care

S0: I have no problem with self-care

S1: I have some problems washing or dressing myself

S2: I am unable to wash or dress myself

Usual activities (e.g., work, study, housework, family or leisure activities)

U0: I have no problem performing my usual activities

U1: I have some problems performing my usual activities

U2: I am unable to perform my usual activities

Pain / Discomfort

PD0: I have no pain or discomfort

PD1: I have moderate pain or discomfort

PD2: I have extreme pain or discomfort

Anxiety / Depression

AD0: I am not anxious or depressed

AD1: I am moderately anxious or depressed

AD2: I am extremely anxious or depressed

b) Total number of patients having each symptom was used as the denominator to calculate the percentage.

c) Following a request from the Japanese Ministry of Health, Labour and Welfare, we removed data from the table if the number of patients corresponding to the data was less than 10.

Table 4 summarizes the results of multivariate logistic regression analyses with each component of QoL as the dependent variable. For all components of QoL, ORs were significantly higher for participants with spinal dysfunction than for those with no spinal dysfunction. For physical health-related components of QoL (i.e., “mobility,” “self-care,” and “usual activities”), ORs were significantly higher for participants with speech dysfunction than for those without. Other than spinal dysfunction and speech dysfunction, the presence of bilateral hearing loss, cerebellar dysfunction, blindness, and hemiparesis was significantly associated with at least one of the physical health-related components of QoL. On the other hand, for subjective and mental health-related components of QoL (i.e., “pain/discomfort” and “anxiety/depression”), other than spinal dysfunction, ORs were significantly higher only for participants with decreased facial sensation and facial nerve palsy as compared with those without these neurological symptoms. Regarding demographic variables, “anxiety/depression” was more deteriorated in participants aged ≤64 years than in those aged ≥65 years.

**Table 4:** Logistic regression analysis using presence/absence of neurological symptoms as the independent variables (n = 187)

**QoL component^a),b)^**	**Mobility**				**Self-care**				**Usual activities**				**Pain / Discomfort**				**Anxiety / Depression**			
					
	**Univariate**		**Multivariate**		**Univariate**		**Multivariate**		**Univariate**		**Multivariate**		**Univariate**		**Multivariate**		**Univariate**		**Multivariate**	
									
	**OR^c)^**	**(95% CI)**	**OR^c)^**	**(95% CI)**	**OR^c)^**	**(95% CI)**	**OR^c)^**	**(95% CI)**	**OR^c)^**	**(95% CI)**	**OR^c)^**	**(95% CI)**	**OR^c)^**	**(95% CI)**	**OR^c)^**	**(95% CI)**	**OR^c)^**	**(95% CI)**	**OR^c)^**	**(95% CI)**

Presence of symptoms (Reference: absence)																				
Hearing loss, unilateral	0.9	(0.5–1.9)	0.3	(0.1–1.1)	1.2	(0.6–2.6)	0.6	(0.2–1.8)	1.3	(0.7–2.6)	1.1	(0.4–2.9)	0.7	(0.4–1.5)	0.5	(0.2–1.2)	1.4	(0.7–2.7)	1.0	(0.5–2.3)
Hearing loss, bilateral	1.4	(0.7–2.9)	0.5	(0.1–1.8)	2.3	(1.1–4.8)	0.6	(0.2–2.1)	3.9	(1.8–8.6)	3.8	(1.3–11.5)	1.7	(0.8–3.6)	1.2	(0.5–3.0)	1.5	(0.8–3.1)	0.8	(0.3–1.9)
Facial nerve palsy	2.5	(1.2–5.3)	2.7	(0.7–10.3)	2.8	(1.4–5.9)	1.8	(0.5–6.1)	2.0	(0.9–4.2)	1.1	(0.3–3.5)	1.2	(0.6–2.4)	0.7	(0.2–1.8)	2.6	(1.3–5.4)	2.7	(1.03–7.0)
Cerebellar dysfunction	9.8	(4.0–23.9)	8.4	(2.0–34.7)	5.4	(2.5–11.4)	1.6	(0.5–5.6)	4.8	(2.0–11.6)	2.7	(0.7–10.4)	1.6	(0.8–3.3)	0.8	(0.3–2.3)	2.4	(1.2–5.0)	0.9	(0.3–2.6)
Decreased facial sensation	3.5	(1.6–7.5)	1.2	(0.4–4.4)	5.0	(2.3–10.8)	3.0	(0.9–9.5)	2.9	(1.3–6.5)	0.8	(0.2–2.6)	5.3	(2.1–13.6)	6.1	(1.9–19.4)	2.3	(1.1–4.8)	1.0	(0.4–2.6)
Speech dysfunction	11.6	(4.5–29.6)	5.0	(1.3–20.1)	7.9	(3.6–17.4)	3.9	(1.2–13.2)	13.5	(4.0–45.9)	9.4	(1.9–46.2)	2.0	(0.95–4.3)	1.2	(0.4–3.5)	3.5	(1.6–7.4)	1.8	(0.6–5.2)
Double vision	2.3	(0.9–6.3)	0.2	(0.1–1.7)	2.4	(0.9–6.5)	0.4	(0.1–2.2)	2.2	(0.8–6.5)	0.4	(0.1–2.3)	2.3	(0.8–6.7)	0.7	(0.2–3.0)	1.9	(0.7–4.9)	0.9	(0.2–3.1)
Blindness	3.9	(1.01–15.4)	18.2	(2.3–146.2)	2.9	(0.8–9.8)	3.8	(0.7–21.5)	3.8	(0.8–18.3)	6.3	(0.9–44.6)	2.3	(0.6–8.9)	2.2	(0.5–10.6)	1.7	(0.5–5.9)	1.7	(0.4–6.9)
Hemiparesis	—	—	—	—	53.1	(6.9–411.1)	53.4	(4.6–613.6)	—	—	—	—	7.5	(1.7–33.8)	4.2	(0.7–27.0)	8.4	(2.3–30.2)	3.3	(0.7–14.7)
Seizures	2.5	(0.7–9.0)	0.3	(0.1–3.7)	4.3	(1.2–15.3)	1.0	(0.1–8.0)	3.8	(0.8–18.3)	1.5	(0.1–16.5)	2.3	(0.6–8.9)	0.9	(0.2–5.1)	2.6	(0.7–9.2)	1.5	(0.3–7.5)
Spinal dysfunction	9.5	(4.8–18.7)	17.9	(6.3–50.6)	6.5	(3.3–12.9)	5.7	(2.3–13.9)	7.3	(3.6–14.6)	8.4	(3.5–20.2)	3.8	(2.0–7.2)	2.8	(1.4–5.8)	3.1	(1.7–5.7)	3.1	(1.5–6.3)
Age (Reference: 0–24 years)																				
25–44 years	1.4	(0.6–3.2)	0.7	(0.2–2.5)	1.6	(0.6–4.0)	0.7	(0.2–2.4)	2.4	(1.04–5.5)	2.0	(0.7–5.9)	1.5	(0.7–3.5)	1.2	(0.5–2.9)	3.5	(1.4–8.9)	3.1	(1.1–8.7)
45–64 years	2.0	(0.8–4.6)	2.1	(0.6–7.4)	1.9	(0.7–4.8)	1.2	(0.4–4.2)	1.7	(0.7–3.8)	1.2	(0.4–3.7)	0.9	(0.4–2.1)	0.7	(0.3–1.7)	3.4	(1.3–8.6)	3.2	(1.1–9.0)
>64 years	1.9	(0.7–5.5)	1.7	(0.4–7.5)	2.4	(0.8–7.4)	2.0	(0.5–8.3)	3.1	(1.1–9.1)	2.3	(0.6–9.8)	1.2	(0.4–3.3)	0.8	(0.3–2.7)	2.0	(0.7–6.3)	1.6	(0.5–5.8)
Sex (Reference: male)	1.1	(0.6–2.0)	0.9	(0.4–2.4)	1.0	(0.5–1.8)	0.8	(0.3–1.8)	0.5	(0.3–1.0)	0.4	(0.2–1.0)	0.9	(0.5–1.6)	1.0	(0.5–2.0)	1.3	(0.7–2.2)	1.2	(0.6–2.3)

a) Classification of each component

Mobility: M0 vs. M1, M2

Self-care: S0 vs. S1, S2

Usual activities: U0 vs. U1, U2

Pain / Discomfort: PD0 vs. PD1, PD2

Anxiety / Depression: AD0 vs. AD1, AD2

b) Reference: M0, S0, U0, PD0, and AD0

c) OR: odds ratio

Appendices 1 and 2 show the results of cross-tabulation and logistic regression analyses between the clinical stage of NF2 and QoL, respectively. As shown in Appendix 2, for all components of QoL, a higher clinical stage was associated with more deteriorated QoL, especially physical health-related components of QoL, although the 95% CIs of ORs were poor due to the relatively small number of participants with moderate/severe impairment of these QoL components. Regarding “pain/discomfort” and “anxiety/depression,” only the highest clinical stage (i.e., Stage 4) was significantly associated with more deteriorated QoL.

## Discussion

This study examined the association between the presence/absence of neurological symptoms of NF2 and deterioration of QoL using a nationwide database of all patients with NF2 who newly claimed for medical expense subsidies in Japan during the past 5 years. The analyses revealed that (1) spinal dysfunction was significantly associated with deterioration of all components of QoL, (2) speech dysfunction and hemiparesis were specifically associated with physical health-related components of QoL, and (3) spinal dysfunction, facial nerve palsy, and age 25–64 years were significantly associated with “anxiety/depression.”

During the 5-year study period, 191 patients with NF2 newly claimed for medical expense subsidies (Fig. 1). Our previous cross-sectional study reported that 409 patients with NF2 submitted claims for subsidies in Japan between 2004 and 2013 [10]. This implies that the number of patients who newly claimed for subsidies for NF2 followed a similar trend pre- and post-enactment of IRDA in Japan.

Nearly 40% of participants had spinal dysfunction (Table 2), and the presence of spinal dysfunction was significantly associated with deterioration of all components of QoL (Table 4). These findings are consistent with a previous study in Japan reporting that, among patients with NF2, those with spinal dysfunction were significantly more likely to experience a loss of social independence compared with those without [11]. Previous studies have reported that spinal tumors are associated with poor prognostic factors among patients with NF2. Patients with spinal tumors were more likely to have more intracranial meningiomas and other intracranial tumors [16]. These deteriorated clinical courses of NF2 may be strongly associated with the impairment of several components of QoL, including mental health-related components.

Speech dysfunction and hemiparesis were specifically associated with physical health-related components of QoL. These findings are consistent with our previous study reporting that patients who had developed speech dysfunction and hemiparesis during the study period were significantly more likely to experience a loss of social independence [11]. Speech dysfunction was associated with physical health-related components of QoL, especially “usual activities.” This may be because the “speech dysfunction” category included dysphagia and aphasia (i.e., a language disorder caused by damage to parts of the brain that control speech) in the present study.

The presence of spinal dysfunction, decreased facial sensation, and facial nerve palsy was significantly associated with subjective and mental health-related components of QoL. Previous studies have shown that neurofibromatosis, including NF2, was associated with symptoms of depression and anxiety, higher levels of stress, and lower levels of self-esteem [17]. Spinal dysfunction, decreased facial sensation, and facial nerve palsy have been reported to be associated with the occurrence of loss of social independence [11], suggesting that patients with these symptoms are more likely to be unemployed and have financial difficulties, which might have led to mental/psychological distress, as represented by the “anxiety/depression” component of QoL.

When adjusted for the presence/absence of neurological symptoms, age 25–64 years was significantly associated with “anxiety/depression” in the multivariate analyses. Compared with those aged ≤24 years or ≥65 years, patients aged 25–64 years who have been diagnosed with NF2 may be more likely to face difficulties in making psychological adjustments due to the disease, such as the failure to fulfill their social roles, unemployment, and financial difficulties.

Regarding clinical stage, for all components of QoL, the highest clinical stage (i.e., Stage 4) was significantly associated with more deteriorated QoL (Appendix 2). Having multiple neurological symptoms could negatively affect both physical and psychological components of QoL in patients with NF2. Although less than 10% of participants were recorded as having severe impairment in each QoL component (Table 1), longitudinal studies will be needed to examine changes in clinical stage and the subsequent deterioration of QoL among patients with NF2.

According to a previous review [1], genes responsible for neurofibromatosis type 1 (NF1) and NF2 are located on different chromosomes as revealed by genetic linkage analysis, and these two disease types have formally been separated. A significant difference was observed in the state of QoL between patients with NF2 in the present study and those with NF1 in our previous study [13], both of which used the nationwide database of patients in Japan during 2015–2019. In particular, the “mobility” and “usual activities” components of QoL appeared to be significantly more deteriorated in patients with NF2 (i.e., present study) than in those with NF1 (i.e., previous study) [13]. This may be due to the fact that most patients with NF2 suffer from deafness and eventually require wheelchair assistance [1].

### Strengths and limitations

The database used in the present study included information on all patients with NF2 who newly submitted medical certificates filled out by physicians to receive medical expense subsidies in Japan during the past 5 years. There are, however, some limitations worth noting. First, patients with NF2 who did not apply for medical expense subsidies during the study period were not included in the study; this could potentially have resulted in selection bias. Second, while a previous study revealed that QoL tended to be more deteriorated in patients with neurofibromatosis than in healthy subjects [18], we could not compare the levels of QoL between patients with NF2 and the general population. Third, as this study was cross-sectional in design, it was not possible to determine the causality between the presence/absence of neurological symptoms and clinical stage and QoL among patients with NF2. Fourth, since the database lacked information on comorbidities, the possibility cannot be ruled out that QoL might have been affected by underlying disorders other than NF2. Fifth, although, to our knowledge, this is the largest epidemiological study on QoL among patients with NF2 using a national database of patients, the relatively small sample size did not allow us to accurately examine the characteristics of QoL among patients with NF2 in multivariate analyses. However, this is inevitable given that NF2 is an intractable “rare” disease. Finally, study participants were limited to patients with NF2 living in Japan. Thus, caution should be exercised when generalizing the present findings to different populations.

## Conclusions

In the present epidemiological study using a national database of NF2 in Japan, 31% to 55% of participants were recorded as having moderate/severe impairment of QoL. Spinal dysfunction was significantly associated with deterioration of all components of QoL, including subjective and mental health-related components of QoL, whereas speech dysfunction and hemiparesis were specifically associated with physical health-related components of QoL.

## Abbreviations

IRDA: Intractable Rare Disease Act
MHLW: Ministry of Health, Labour and Welfare
NF1: Neurofibromatosis type 1
NF2: Neurofibromatosis type 2
QoL: Quality of life

## Supplementary information

**Additional file 1:** **Appendix 1.** Cross-tabulation between clinical stage of NF2 and quality of life (QoL) (n = 187). **Appendix 2.** Logistic regression analysis using clinical stage as the independent variable (n = 187).

## Declarations

### Data availability

Data cannot be shared for privacy or ethical reasons.
