# Supplementary material for: Quality of life and neurological symptoms in patients with neurofibromatosis type 2: a national database study in Japan
Source: Environ Health Prev Med. 2024 Aug 28;29:44. doi: 10.1265/ehpm.24-00158 (PMC11362668; doi:10.1265/ehpm.24-00158)
Supplement: Supplementary file 1 — Additional file 1: Appendix 1. Cross-tabulation between clinical stage of NF2 and quality of life (QoL) (n = 187). Appendix 2. Logistic regression analysis using clinical stage as the independent variable (n = 187). [file ehpm-29-044-s001.docx]

| Appendix 1. Cross-tabulation between clinical stage of NF2 and quality of life (QoL) (n=187) | | | | | | | | | | | | | | | | | |
| --- | --- | --- | --- | --- | --- | --- | --- | --- | --- | --- | --- | --- | --- | --- | --- | --- | --- |
| QoL component ^a)^ | Mobility | | | | |  | Self-care | | | | |  | Usual activities | | | | |
|  | M0 | |  | M1/M2 | |  | S0 | |  | S1/S2 | |  | U0 | |  | U1/U2 | |
|  | n | (%) |  | n | (%) |  | n | (%) |  | n | (%) |  | n | (%) |  | n | (%) |
| Clinical stage |  | | | | |  |  | | | | |  |  | | | | |
| Stage 0 (n=32) | — |  |  | — |  |  | — |  |  | — |  |  | — |  |  | — |  |
| Stage 1 (n=21) | — |  |  | — |  |  | — |  |  | — |  |  | — |  |  | — |  |
| Stage 2 (n=35) | 21 | (60) |  | 14 | (40) |  | 25 | (71) |  | 10 | (29) |  | 16 | (46) |  | 19 | (54) |
| Stage 3 (n=11) | — |  |  | — |  |  | — |  |  | — |  |  | — |  |  | — |  |
| Stage 4 (n=88) | 31 | (35) |  | 57 | (65) |  | 44 | (50) |  | 44 | (50) |  | 19 | (22) |  | 69 | (78) |
|  |  |  |  |  |  |  |  |  |  |  |  |  |  |  |  |  |  |
| Appendix 1. Cross-tabulation between clinical stage of NF2 and quality of life (QoL) (n=187) (continued) | | | | | | | | | | | |  |  |  |  |  |  |
| QoL component ^a)^ | Pain / Discomfort | | | | |  | Anxiety / Depression | | | | |  |  |  |  |  |  |
|  | PD0 | |  | PD1/PD2 | |  | AD0 | |  | AD1/AD2 | |  |  |  |  |  |  |
|  | n | (%) |  | n | (%) |  | n | (%) |  | n | (%) |  |  |  |  |  |  |
| Clinical stage |  | | | | |  |  | | | | |  |  |  |  |  |  |
| Stage 0 (n=32) | 19 | (59) |  | 13 | (41) |  | — |  |  | — |  |  |  |  |  |  |  |
| Stage 1 (n=21) | — |  |  | — |  |  | — |  |  | — |  |  |  |  |  |  |  |
| Stage 2 (n=35) | 16 | (46) |  | 19 | (54) |  | 22 | (63) |  | 13 | (37) |  |  |  |  |  |  |
| Stage 3 (n=11) | — |  |  | — |  |  | — |  |  | — |  |  |  |  |  |  |  |
| Stage 4 (n=88) | 26 | (30) |  | 62 | (70) |  | 38 | (43) |  | 50 | (57) |  |  |  |  |  |  |
| a) Classification of each component Mobility M0: I have no problem walking about M1: I have some problems walking about M2: I am confined to bed Self-care S0: I have no problem with self-care S1: I have some problems washing or dressing myself S2: I am unable to wash or dress myself Usual activities (e.g., work, study, housework, family or leisure activities) U0: I have no problem performing my usual activities U1: I have some problems performing my usual activities U2: I am unable to perform my usual activities Pain / Discomfort PD0: I have no pain or discomfort PD1: I have moderate pain or discomfort PD2: I have extreme pain or discomfort Anxiety / Depression AD0: I am not anxious or depressed AD1: I am moderately anxious or depressed AD2: I am extremely anxious or depressed  b) Total number of patients having each symptom was used as the denominator to calculate the percentage.  c) Following a request from the Japanese Ministry of Health, Labour and Welfare, we removed data from the table if the number of patients corresponding to the data was less than 10. | | | | | | | | | | | | | | | | | |

| Appendix 2. Logistic regression analysis using clinical stage as the independent variable (n=187) | | | | | | | | | | | | | | |
| --- | --- | --- | --- | --- | --- | --- | --- | --- | --- | --- | --- | --- | --- | --- |
| QoL component ^a),b)^ | Mobility | |  | Self-care | |  | Usual activities | |  | Pain/Discomfort | |  | Anxiety/Depression | |
|  | OR ^c)^ | (95% CI) |  | OR ^c)^ | (95% CI) |  | OR ^c)^ | (95% CI) |  | OR ^c)^ | (95% CI) |  | OR ^c)^ | (95% CI) |
| Clinical stage (Reference: Stage 0) |  |  |  |  |  |  |  |  |  |  |  |  |  |  |
| Stage 1 | — | — |  | — | — |  | 1.9 | (0.4-8.6) |  | 0.7 | (0.2-2.3) |  | 1.3 | (0.3-5.0) |
| Stage 2 | 4.6 | (1.3-16.2) |  | 12.8 | (1.5-107.8) |  | 9.6 | (2.7-35.0) |  | 1.8 | (0.7-5.1) |  | 2.6 | (0.8-8.4) |
| Stage 3 | 4.0 | (0.8-20.4) |  | 11.8 | (1.1-130.3) |  | 13.2 | (2.5-70.1) |  | 0.3 | (0.1-1.8) |  | 2.3 | (0.5-10.7) |
| Stage 4 | 13.4 | (4.2-43.2) |  | 31.1 | (4.0-242.4) |  | 25.2 | (7.6-83.9) |  | 3.8 | (1.6-9.1) |  | 5.5 | (2.0-15.3) |
| Age (Reference: 0-24 years) |  |  |  |  |  |  |  |  |  |  |  |  |  |  |
| 25-44 years | 0.9 | (0.3-2.5) |  | 1.1 | (0.4-3.2) |  | 2.3 | (0.8-6.3) |  | 1.3 | (0.5-3.2) |  | 2.9 | (1.1-7.7) |
| 45-64 years | 1.2 | (0.4-3.2) |  | 1.1 | (0.4-3.2) |  | 1.1 | (0.4-2.8) |  | 0.7 | (0.3-1.7) |  | 2.5 | (0.9-6.7) |
| >64 years | 0.9 | (0.3-2.9) |  | 1.2 | (0.3-4.4) |  | 2.1 | (0.5-8.2) |  | 0.7 | (0.2-2.2) |  | 1.3 | (0.4-4.2) |
| Sex (Reference: male) | 1.3 | (0.6-2.5) |  | 1.1 | (0.6-2.3) |  | 0.5 | (0.3-1.1) |  | 0.9 | (0.5-1.7) |  | 1.4 | (0.7-2.6) |
| a) Classification of each component Mobility: M0 vs. M1,M2 Self-care: S0 vs. S1,S2 Usual activities: U0 vs. U1,U2 Pain / Discomfort: PD0 vs. PD1,PD2 Anxiety / Depression: AD0 vs. AD1,AD2 b) Reference: M0, S0, U0, PD0, and AD0 c) OR: odds ratio | | | | | | | | | | | | | | |
